# Affective Go/NoGo fMRI study of adults living with ADHD reveals differential BOLD activation in ACC, insula, and secondary visual cortex

**DOI:** 10.1192/j.eurpsy.2025.290

**Published:** 2025-08-26

**Authors:** J. M. Réthelyi, M. Baradits, B. Kakuszi, P. Czobor

**Affiliations:** 1 Department of Psychiatry and Psychotherapy, Semmelweis University, Budapest, Hungary

## Abstract

**Introduction:**

Attention-Deficit/Hyperactivity Disorder (ADHD) is a neurodevelopmental disorder that begins in childhood and persists into adulthood in 35-50% of cases, resulting in a prevalence of 2-4% in the adult population. The core symptoms are inattention, hyperactivity, and impulsivity, while emotional dysregulation is considered an associated symptom. Several lines of evidence support structural and functional brain imaging alterations in ADHD. Decreased volume of the accumbens and amygdala persist from childhood into adulthood, and several resting-state and task-related functional alterations have also been reported.

**Objectives:**

To investigate differential brain activation patterns in adults diagnosed with ADHD as compared to healthy controls (HC) during an affective Go/NoGo task, to understand the neural background of selective inhibition in emotionally demanding situations.

**Methods:**

Data of 61 subjects were analyzed: 34 adult ADHD patients meeting DSM-IV diagnostic criteria (17 women, 17 men, mean age 37.3 years) and 19 HCs (10 women, 9 men, mean age 30.2 years). HCs were screened using the 90-item Symptom Checklist to exclude current psychiatric comorbidity. Symptom severity was assessed using the Conners’ Adult ADHD Rating Scale (CAARS). During the MRI scan in a Philips Achieva 3.0T scanner, we used a block design delivering positive, negative and neutral stimuli from the IAPS System applying a Go/NoGo paradigm. The fMRI analysis focused on contrasts between task and rest, emotional and neutral, and Go and NoGo conditions (activation or deactivation). Contrast between activation and deactivation in the ADHD and HC groups were tested.

**Results:**

Based on the pooled data from both groups, we found significant contrasts between the task and rest conditions, including an activation of primary and secondary visual cortex, frontal areas, corresponding to the Visual Network (VN), the Dorsal- and Ventral- Attention Networks (DAN, VAN), and the Central Executive Network; and a deactivation of the anterior cingulate, precuneus and parietal areas, corresponding to the Default Mode Network (DMN). During emotional vs neutral conditions, we detected activation of the secondary visual cortex, while contrasts between NoGo vs Go conditions manifested as activation of ACC and insular regions. ADHD subjects showed increased BOLD activation in the DAN and VAN areas. In all conditions, DMN areas showed higher deactivation in the ADHD group.

**Conclusions:**

Here, we report differences in VN, DAN and VAN brain regions and the DMN in adult ADHD patients suggestive of distinctive pattern of activation and deactivation during an emotional Go/NoGo task. The results have clinical implications for understanding ADHD patients under in emotionally demanding situations.

Funding: Hungarian Brain Research Program, grants 2017-1.2.1-NKP-2017-00001 ans #NAP2022-I-4/2022 to JMR and PC.

**Disclosure of Interest:**

None Declared

